# Bortezomib, Ifosfamide, Carboplatin, and Etoposide in a Patient with HIV-Negative Relapsed Plasmablastic Lymphoma

**DOI:** 10.1155/2016/3598547

**Published:** 2016-11-13

**Authors:** Mehmet Akce, Elaine Chang, Mohammad Haeri, Mike Perez, Christie J. Finch, Mark M. Udden, Martha P. Mims

**Affiliations:** ^1^Department of Medicine, Section of Hematology-Oncology, Baylor College of Medicine, Houston, TX 77030, USA; ^2^Department of Pathology, Baylor College of Medicine, Houston, TX 77030, USA

## Abstract

Plasmablastic lymphoma (PBL) is a rare subtype of diffuse large B cell lymphoma (DLBCL), often associated with HIV infection. We present a case of a 53-year-old HIV-negative man with untreated hepatitis C viral infection who presented with abdominal pain and lymphadenopathy. Lymph node and bone marrow biopsies were consistent with plasmablastic lymphoma. He had partial response (PR) to 6 cycles of EPOCH but disease progressed seven weeks later. Repeat biopsy was consistent with plasmablastic lymphoma. Three cycles of bortezomib, ifosfamide, carboplatin, and etoposide (B-ICE) chemotherapy resulted in a partial response (PR). Five months later, he presented with widespread lymphadenopathy and tumor lysis syndrome with circulating blasts. Flow cytometry revealed a different population of lymphoma cells, this time positive for CD5, CD19, CD20, and CD22, with dim expression of CD45 and CD38. The patient died on the first day of ESHAP chemotherapy. There are no treatment recommendations or standard of care for plasmablastic lymphoma. A literature search yielded 10 cases in which bortezomib was administered in either HIV-positive or HIV-negative PBL. Six reported a partial response, 3 reported a complete response, and 1 was a near-complete response. Bortezomib, in combination with chemotherapy, may be an effective treatment option in PBL as reported here.

## 1. Introduction

Plasmablastic lymphoma (PBL) is a rare and aggressive subtype of diffuse large B cell lymphoma (DLBCL), first described in HIV patients [1–3]. PBL comprises an estimated 2% of the HIV-related lymphomas. In HIV-negative patients, PBL is associated with increased age or immunosuppression. Though increasingly recognized in recent years, HIV-negative PBL remains a rare diagnosis [2]. HIV-negative PBL shares some diagnostic and therapeutic challenges with HIV-positive PBL, including lack of CD20 expression and early chemotherapy relapse. Standard cyclophosphamide, doxorubicin, vincristine, and prednisone (CHOP) chemotherapy is thought to be inadequate for the treatment of PBL, yet there is no standard treatment recommendation for these patients. Here, we present a case of HIV-negative PBL in which significant response to B-ICE chemotherapy regimen was observed in the second-line setting.

## 2. Case Presentation

**Figure 1:**
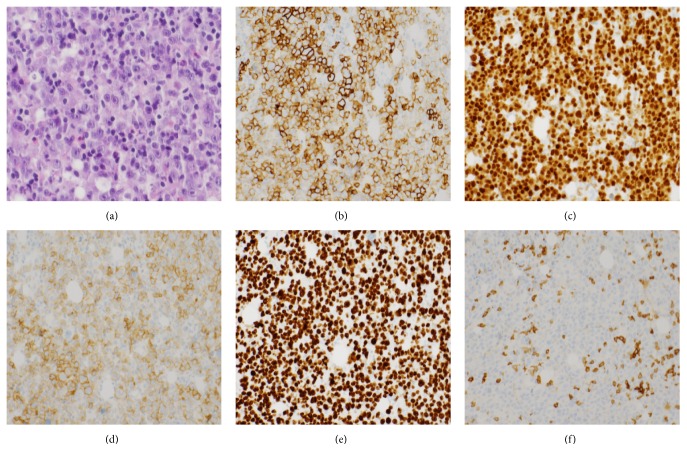
(a) Plasmablastic lymphoma, with immunoblastic morphology, involving the bone marrow. (HE stain) (b, c) the neoplastic cells are positive for CD138 and MUM-1 respectively, indicating the plasma cell origin of this neoplasm. (d) The neoplastic cells are positive for CD30. (e) Strong and homogenous staining with Ki-67 indicating a high proliferation index. (f) The neoplastic cells are negative for CD5. Few, scattered T cells staining positive for CD5 are identified.

**Figure 2:**
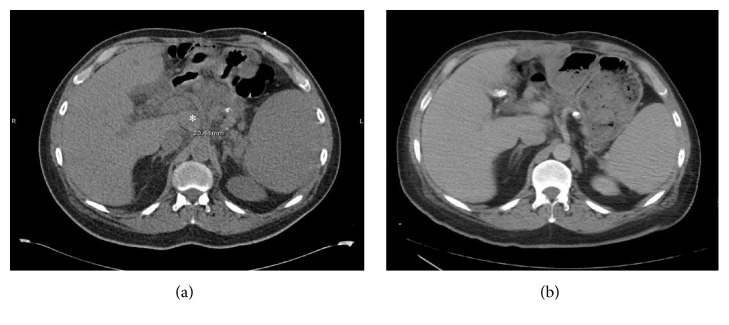
(a) CT without contrast. Pretreatment lymph node (*∗*) is 2 cm. (b) CT with contrast. Posttreatment lymph node on a comparable level is not visible.

**Figure 3:**
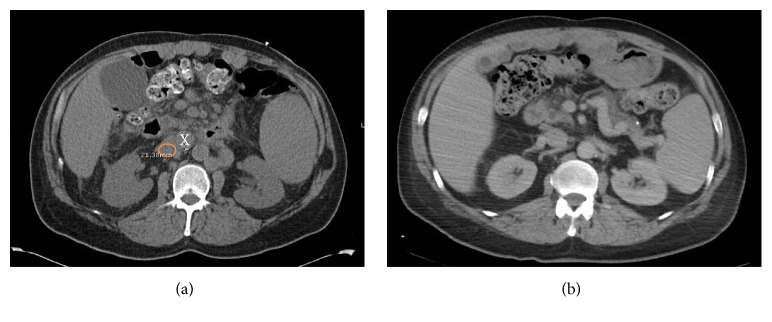
(a) CT without contrast. Pretreatment lymph node (X) is 2.1 cm. Orange circle is IVC. (b) CT with contrast. Posttreatment lymph node on a comparable level is not visible.

A 53-year-old man with untreated chronic hepatitis C virus (HCV) infection presented with right lower quadrant abdominal pain. Initial laboratory examination revealed a white blood cell (WBC) count of 10,700 cells/mm^2^, hemoglobin 9.6 g/dL, platelets 82,000/*μ*L, creatinine 1 mg/dL, total protein 7.4 g/dL, albumin 3.6 g/dL, lactate dehydrogenase (LDH) 7347 U/L, and uric acid 6.6 mg/dL. HIV serologies were negative. Computerized tomography (CT) of the chest/abdomen/pelvis (C/A/P) with contrast revealed new extensive supra- and infradiaphragmatic lymphadenopathy and splenomegaly (17.6 cm). Immunohistochemical (IHC) staining of a lymph node core biopsy was positive for CD138, CD30, CD45 (weak), MUM-1, PAX-5 (weak), BCL-6 (weak), Ki-67 > 90%, and EMA (very rare) and negative for CD3, CD5, CD10, BCL-2, CD21, CD20, ALK, CD117, CD79a, CD56, Cyclin D1, and HHV-8. Epstein Barr virus was not detected by either EBER or EBV-LMP stains. Flow cytometry detected a kappa light chain restricted population with plasmacytic differentiation positive for CD45, CD38, surface and cytoplasmic (s/c) kappa, and CD8 (subset) and negative for CD19, CD20, CD22, CD79a, CD56, and s/c lambda, consistent with PBL. Bone marrow examination revealed heavy involvement by PBL, with 36% plasmablasts and an IHC profile similar to the lymph node biopsy (Figure 1). Marrow cytogenetics revealed a normal male karyotype. EPOCH (etoposide, prednisone, vincristine, cyclophosphamide, and doxorubicin) was given for 3 cycles. Doxorubicin was subsequently omitted because of mild reduction of the left ventricular ejection fraction. After a total of 6 cycles, CT scans showed partial response (PR). Seven weeks after completion of therapy, the patient presented with severe back pain. Laboratory results included WBC count of 6,300 cells/*μ*L, hemoglobin 9.3 g/dL, platelets 56,000/*μ*L, creatinine 1.2 mg/dL, LDH 3430 U/L, and uric acid 13.4 mg/dL. CT imaging revealed progression of disease with widespread lymphadenopathy. Although a lymph node specimen was unsatisfactory due to scant cellularity, bone marrow biopsy was consistent with plasmablastic lymphoma. B-ICE (bortezomib [1.3 mg/m^2^ days 1 and 4 subcutaneously], ifosfamide [5000 mg/m^2^ day 2 IV], carboplatin [area under the curve (AUC) 5 on day 2 IV], and etoposide [100 mg/m^2^ days 1–3 IV every 3 weeks]) was initiated. Repeat CT imaging after three cycles showed complete resolution of lymph nodes (Figures 2 and 3). Therefore, maintenance bortezomib 1.3 mg/m^2^ subcutaneous every 2 weeks was initiated. Bortezomib was held after 4 doses due to grade 3 neuropathy. Seven weeks after the last dose of bortezomib, positron emission tomography (PET)/CT scan showed disease progression, with worsening splenomegaly (18 cm) and diffuse lymphadenopathy. Biopsy was delayed due to CHF exacerbation and decline in performance status.

**Figure 4:**
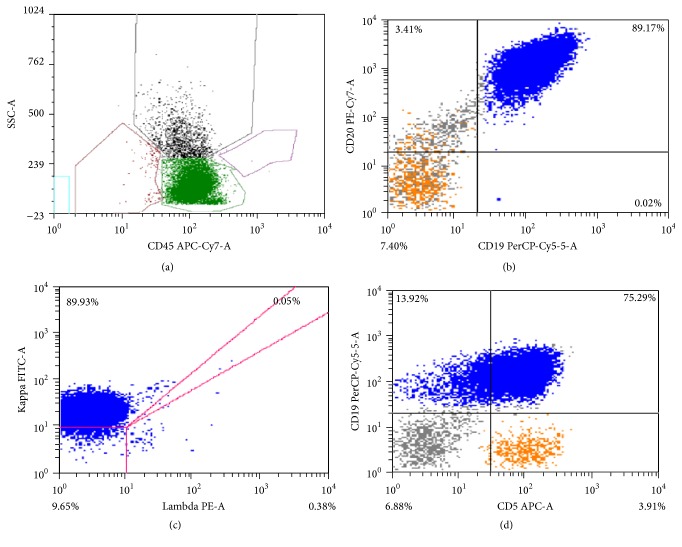
(a) Flow cytometry, side scatter versus CD45, demonstrates a large, atypical lymphocyte population shifted into the blast/myeloid gate. (b) CD19 versus CD20 evaluation identifies a CD19/CD20 positive B lymphocyte population. (c) Surface kappa light chain restriction is indicative of monoclonal B lymphocyte population. (d) The neoplastic B cells are positive for CD19 and CD5. The previous plasmablastic neoplasm identified in this patient was CD20 and CD5 negative.

**Figure 5:**
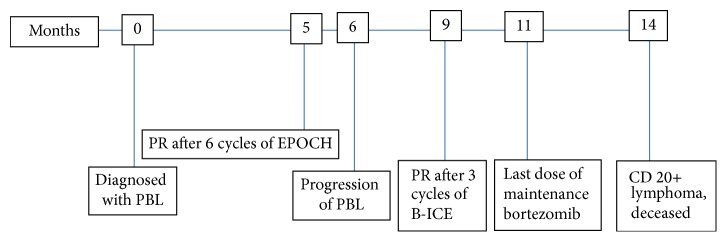
Timeline of clinical course.

Two months later, the patient presented to the hospital with diffuse abdominal pain, elevated WBC count, and acute kidney injury: WBC count of 100,000 cells/mm^2^, hemoglobin 4.2 g/dL, creatinine 1.7 mg/dL, uric acid 11.1 mg/dL, potassium 4.8 mmol/L, phosphorus 2.4 mg/dL, calcium 9.4 mg/dL, and LDH 2374 U/L. CT of chest demonstrated progression of disease as compared to PET/CT with increase in size of multiple mediastinal, axillary, retropectoral, and upper abdominal lymph nodes. Flow cytometry of peripheral blood revealed a surface kappa immunoglobulin light chain CD5-positive B cell population positive for CD19, CD20, CD22, and dim expression of CD45 and CD38, consistent with B cell non-Hodgkin lymphoma (Figure 4). Dexamethasone 20 mg IV twice daily reduced the WBC count to 34,000 cells/*μ*L after 4 days of therapy. ESHAP (etoposide, methylprednisolone, high-dose cytarabine, and cisplatin) was initiated. At the end of the first day of ESHAP, the patient became confused with glucose 421 mg/dL, potassium level of 8.3 mmol/L, and metabolic acidosis with anion gap of 18, thought to be due to diabetic ketoacidosis. Despite treatment for these metabolic derangements, his mental and respiratory status further deteriorated. Based on his stated wishes, comfort care measures were offered and he died within 12 hours. The clinical course, therapies, and responses are outlined in the timeline figure (Figure 5).

## 3. Discussion

HIV-negative PBL, in comparison to HIV-associated PBL, occurs in older patients, with less frequent involvement of oral mucosa or bone marrow and less frequently stages III-IV [4, 5]. A review of 114 HIV-negative patients with PBL, by Liu et al. [4], demonstrated that immunosuppression, stage IV disease, and EBV negativity were poor prognostic factors. Overall survival (OS) is between 9 and 19 months in HIV-negative patients [4, 5]. The patient described here survived 14 months. As PBL is still relatively rare, standard therapy has not been established. CHOP is considered inadequate therapy for the treatment of HIV-positive PBL, and suggested regimens include infusional etoposide, prednisone, vincristine, cyclophosphamide, and doxorubicin (EPOCH), cyclophosphamide, vincristine, doxorubicin, and methotrexate alternating with ifosfamide, etoposide, and cytarabine (CODOX-M/IVAC), or hyperfractionated cyclophosphamide, vincristine, doxorubicin, and dexamethasone alternating with methotrexate and cytarabine (hyper-CVAD). No specific treatment recommendations are available for HIV-negative PBL [6]. In spite of recommendations for intensive regimens, two retrospective studies of HIV-associated PBL patients did not demonstrate benefit with more intensive chemotherapy regimens [7, 8]. One ongoing phase II study is evaluating the safety and effectiveness of dose-adjusted EPOCH+/− rituximab in patients with high-risk DLBCL including PBL (NCT01092182). Another actively recruiting phase II study is evaluating dose-adjusted EPOCH+/− vorinostat in HIV-related DLBCL or other aggressive B cell lymphomas including PBL (NCT01193842).

The benefit of autologous stem cell transplantation (ASCT) in patients with PBL is not clear based on the limited data available. In a single institution report of 9 consecutive HIV-negative PBL patients, 7 patients achieved CR and 1 patient achieved PR after chemotherapy [9]. Four of these 8 patients received ASCT in first CR or PR, and 2 out of 4 transplanted patients were alive at the end of follow-up (median 23.9 months). The third patient developed recurrence at 14 months and the fourth patient developed disease recurrence at 2 months after transplant. In comparison, of the 4 patients who achieved CR and were not transplanted, 3 were alive with no evidence of disease at follow-up and 1 was alive with recurrence. Thus, overall, both groups of patients transplanted or not, had relatively good outcomes in comparison to other reports [9].

In this case report, PR was observed in relapsed PBL when bortezomib was administered in combination with multiagent chemotherapy. Case reports suggest that bortezomib in combination with regimens such as EPOCH can be effective in HIV-associated and HIV-negative PBL and seems promising for long remissions [10–17]. Bortezomib, a proteasome inhibitor, targets the hypothesized pathogenesis of PBL, deficient apoptosis of plasmablasts. The plasmablast is normally a short-lived, antibody-secreting cell that frequently undergoes apoptosis in the germinal center. Incompletely understood mechanisms protect cancerous plasmablasts from apoptosis. Proteasomal inhibition appears to be the primary mechanism through which bortezomib exerts its action, but other possibilities also exist. Through phosphorylation of bcl-2, bortezomib induces G2-M phase cell cycle arrest, resulting in apoptosis of malignant cells [18]. Additionally, bortezomib induces oxidative and endoplasmic reticulum stress, thus making it particularly toxic to secretory cells characterized by high protein production, such as PBL cells [15–19]. This is demonstrated clinically in multiple myeloma, where bortezomib sensitivity correlates with the amount of immunoglobulin production [20]. The use of bortezomib in the PBL literature has ranged from monotherapy to combination therapy and from first line to fourth line [10–17]. Among the 10 cases reported in the literature, 4 cases were in HIV-positive patients and 6 cases were in HIV-negative ones. Six reported a partial response (PR), 3 reported a CR, and 1 was a near-complete response. The onset of response was almost universally immediate, and the duration of response was between 2 and 24 months. Many have found an acceptable toxicity profile, even for patients with poor performance status.

The patient reported here relapsed with aggressive disease including circulating blasts not matching the original disease. Interestingly these circulating blasts were positive for CD19, CD20, and CD22 by flow cytometry. Transformation of PBL to another lymphoma from PBL has been reported by Bibas et al. In that case, the patient achieved a PR following a bortezomib-containing regimen given in the second-line setting but relapsed with “PBL with morphologic Burkitt-like features” [10]. It is unclear whether bortezomib had any role in transformation to another lymphoma in either case, but no other cases of transformation have been reported.

## 4. Conclusion

We report the use of B-ICE in relapsed PBL in an HIV-negative patient with poor prognostic factors (stage IV and EBV-negative disease). Though brief, the patient achieved PR and complete resolution of lymph nodes by CT imaging and OS of 14 months. Given the lack of standard treatment, B-ICE may expand treatment options in PBL and serve as a bridge to ASCT in eligible patients. Due to transient response and worsened comorbidities ASCT was not entertained for our patient. The role of bortezomib has not been fully delineated, but so far the depth and rapidity of response are promising. Prospective trials of bortezomib, alone or in combination with other regimens, are needed to define the role of bortezomib in PBL.
